# Chaudhuri’s Dashboard of Vitals in Parkinson’s syndrome: an unmet need underpinned by real life clinical tests

**DOI:** 10.3389/fneur.2023.1174698

**Published:** 2023-05-25

**Authors:** Mubasher A. Qamar, Silvia Rota, Lucia Batzu, Indu Subramanian, Cristian Falup-Pecurariu, Nataliya Titova, Vinod Metta, Iulia Murasan, Per Odin, Chandrasekhara Padmakumar, Prashanth L. Kukkle, Rupam Borgohain, Rukmini Mridula Kandadai, Vinay Goyal, Kallol Ray Chaudhuri

**Affiliations:** ^1^Institute of Psychiatry, Psychology and Neuroscience, Department of Basic and Clinical Neuroscience, Division of Neuroscience, King’s College London, London, United Kingdom; ^2^King’s College Hospital NHS Foundation Trust, London, United Kingdom; ^3^Department of Neurology, David Geffen School of Medicine, University of California, Los Angeles, Los Angeles, CA, United States; ^4^Parkinson’s Disease Research, Education and Clinical Centers, Greater Los Angeles Veterans Affairs Medical Center, Los Angeles, CA, United States; ^5^Faculty of Medicine, Transilvania University of Braşov, Brașov, Romania; ^6^Department of Neurology, County Clinic Hospital, Brașov, Romania; ^7^Department of Neurology, Neurosurgery and Medical Genetics, Federal State Autonomous Educational Institution of Higher Education “N.I. Pirogov Russian National Research Medical University” of the Ministry of Health of the Russian Federation, Moscow, Russia; ^8^Department of Neurodegenerative Diseases, Federal State Budgetary Institution “Federal Center of Brain Research and Neurotechnologies” of the Federal Medical Biological Agency, Moscow, Russia; ^9^Department of Neurology, University Hospital, Lund, Sweden; ^10^Older Person’s Medical Clinic, Broadmeadow, NSW, Australia; ^11^Center for Parkinson’s Disease and Movement Disorders, Manipal Hospital, Karnataka, India, Bangalore; ^12^Parkinson’s Disease and Movement Disorders Clinic, Bangalore, Karnataka, India; ^13^Department of Neurology, Nizam’s Institute of Medical Sciences, Autonomous University, Hyderabad, India; ^14^Neurology Department, Medanta, Gurugram, India

**Keywords:** Parkinson’s disease, motor, nonmotor, vitals, dashboard, oral, gut, vision

## Abstract

We have recently published the notion of the “vitals” of Parkinson’s, a conglomeration of signs and symptoms, largely nonmotor, that must not be missed and yet often not considered in neurological consultations, with considerable societal and personal detrimental consequences. This “dashboard,” termed the Chaudhuri’s vitals of Parkinson’s, are summarized as 5 key vital symptoms or signs and comprise of (a) motor, (b) nonmotor, (c) visual, gut, and oral health, (d) bone health and falls, and finally (e) comorbidities, comedication, and dopamine agonist side effects, such as impulse control disorders. Additionally, not addressing the vitals also may reflect inadequate management strategies, leading to worsening quality of life and diminished wellness, a new concept for people with Parkinson’s. In this paper, we discuss possible, simple to use, and clinically relevant tests that can be used to monitor the status of these vitals, so that these can be incorporated into clinical practice. We also use the term Parkinson’s syndrome to describe Parkinson’s disease, as the term “disease” is now abandoned in many countries, such as the U.K., reflecting the heterogeneity of Parkinson’s, which is now considered by many as a syndrome.

## Introduction

**Figure 1:**
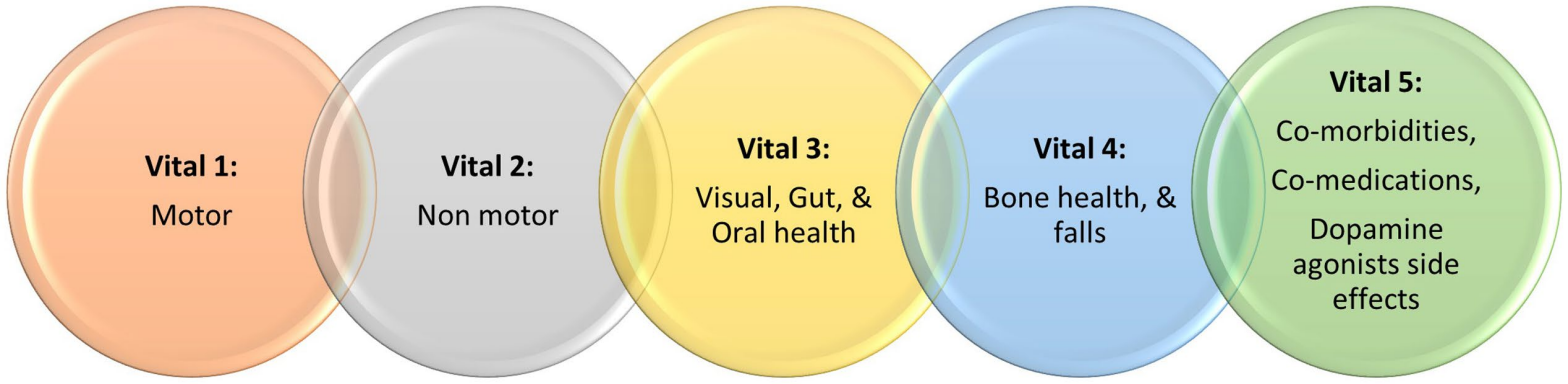
A summary of the Chaudhuri’s Dashboard of Vitals in Parkinson’s, adapted from Chaudhuri et al. (6).

First described by James Parkinson’s in 1817 (1), Parkinson’s disease (PD), which should be regarded as a syndrome, is a progressive neurodegenerative condition, whose incidence has risen sharply worldwide, leading some authors to suggest we may be facing a Parkinson’s pandemic (2). Modern concepts of PD (3) extend beyond the well characterized motor symptoms to address nonmotor symptoms (NMS) (4), as well as to include the concept of wellness (5), the latter a marriage of motor, nonmotor, socio-cultural and socio-economic issues. Taking these into account, we recently proposed the concept of the “vitals” of Parkinson’s (6), which are comprised of the crucially important comorbid or related symptoms of PD. Vitals consist of well-established essential motor and nonmotor features of PD, but also include other health factors that are crucial to providing holistic care, encompassing patients’ safety and quality of life (QoL). Often motor and NMS can occupy an entire consultation, however we bring to attention and stress on the importance of three other vitals (Figure 1), which are often omitted by healthcare professionals and rarely reported by people with Parkinson’s (PwP). When these vitals are missed in clinical practice, it can lead to marked deterioration of patients’ wellness. According to our and our collaborators’ clinical experience across sites in the U.K., U.A.E., and Romania, we have seen limited usage of these vitals even in well-established PD clinics, with consequences such as poor absorption of levodopa (7), or fractures secondary to falls due to non-detection of osteoporosis (8), or potentially dangerous daytime sleepiness and sudden onset of sleep (9).

**Figure 2:**
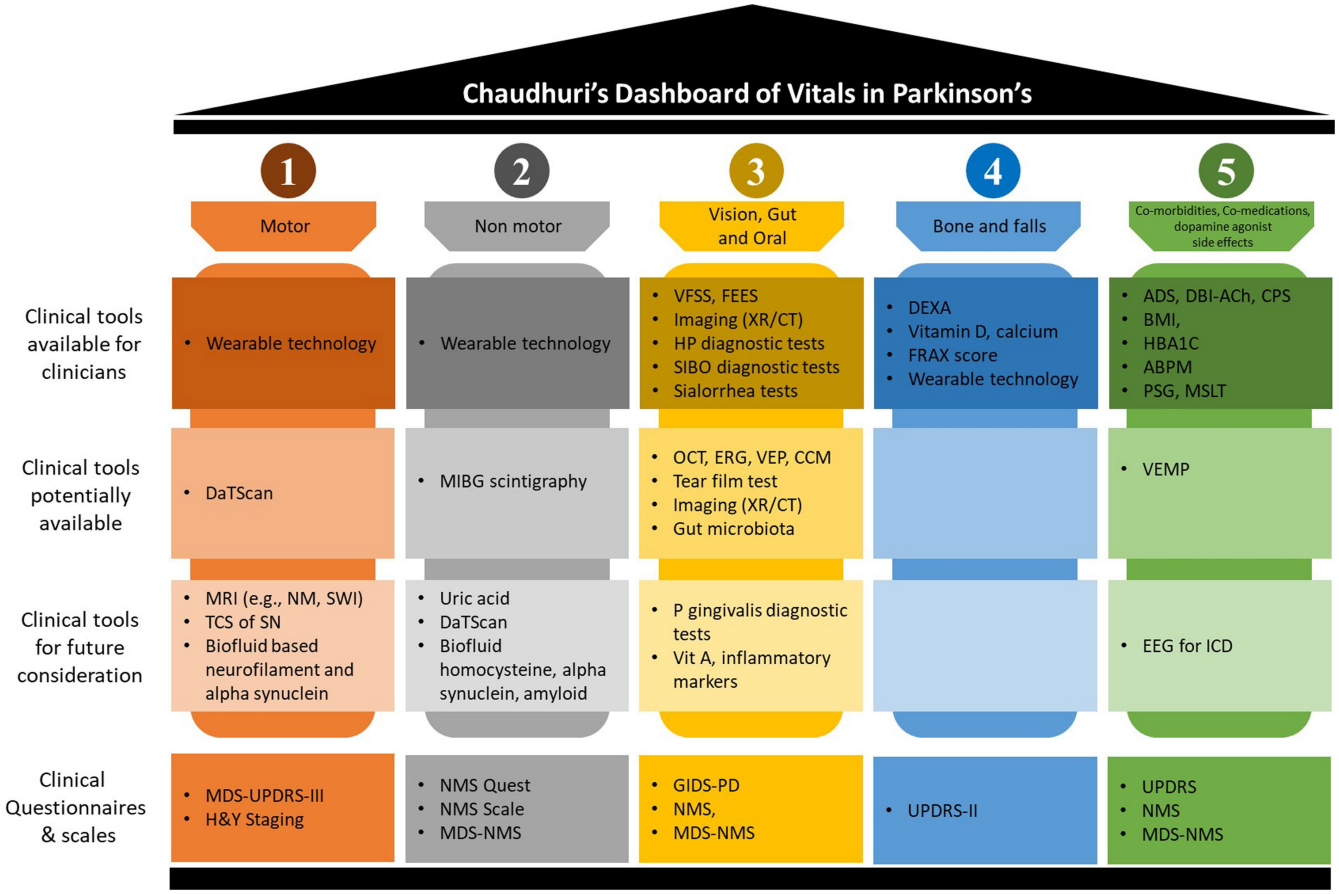
Chaudhuri’s Dashboard of Vitals clinical tools. This figure summarizes discussed clinical tools when applied to the Dashboard of Vitals in Parkinson’s disease. The figure splits the tools into four categories; (1) clinical tools available for clinicians, these are currently available tools with good evidence to application to real-clinical practice; (2) clinical tools potentially available, these are tools which may limited due to resources and should be considered with careful consideration depending on the clinical indications; (3) clinical tools for future consideration, these are tools which may be available or currently being developed but are not solely able to be applied without further evidence for their application in clinical practice; and (4) clinical questionnaires and scales, these are tools which allow for screening and progression monitoring in clinical practice. ABPM, ambulatory blood pressure monitor; ADS, anticholinergic drug scale; BMI, body mass index; CCM, corneal confocal microscopy; CPS, co-morbidities polypharmacy scale; CT, computerized tomography; DaTScan, presynaptic dopamine transporter single-photon emission computerized tomography; DBI-Ach, burden index anticholinergic component; DEXA, dual-energy X-ray absorptiometry; EEG, electroencephalogram; ERG, electroretinography; FEES, fibreoptic endoscopic evaluation of swallowing; FRAX, fracture risk assessment tool; GIDS, gastrointestinal dysfunction scale; H&Y, Hoehn and Yahr; HBA1C, glycated hemoglobin; HP, helicobacter pylori; ICD, impulsive control disorder; MDS, movement disorder society; MIBG, 123I-metaiodobenzylguandine; MRI, magnetic resonance imaging; MSLT, multiple sleep latency tests; NM, neuromelanin; NMS, nonmotor symptoms; OCT, optical coherence tomography; P gingivalis, porphyromonas gingivalis; PD, Parkinson’s disease; PSG, polysomnography; QUEST, questionnaire; SIBO, small intestinal bacterial overgrowth; SN, substantia nigra; SWI, susceptibility weighted imaging; TCS, transcranial sonography; UPDRS, unified Parkinson’s disease rating scale; VEMP, vestibular-evoked myogenic potential; VEP, visual evoked potential; VFSS, videofluoroscopic swallowing study; Vit, vitamin; XR, x-ray.

Adopting the new “Chaudhuri’s Dashboard of Vitals in Parkinson’s” as a blueprint (Figure 1), in this review we discuss the tests that can be either be used or have the potential to be used once properly validated, in everyday clinical practice to assess these vitals. Some of clinical tools discussed are already in use for other conditions or clinical purposes, while others are more investigational at present. Figure 2 summarize the clinical tools we discuss in this review, divided into three categories according to the current applicability. It also includes useful clinical scales and questionnaires, which are beyond the scope of this review.

## Dashboard vital: motor

The cardinal motor features of PD include bradykinesia, resting tremor, rigidity, and postural instability. Our understanding of PD has evolved to appreciate there are different motor subtypes, such as tremor-dominant (TD), postural instability and gait-disturbance (PIGD), akinetic-rigid (AR) subtypes, allowing for a more personalized approach in the clinical practice (10). These motor phenotypes can be differentiated through algorithms developed from the Unified Parkinson’s Disease Rating Scale (UPDRS) (11) or the revised Movement Disorder Society (MDS-UPDRS) (12), or with the use of specific motor symptoms scales such as the Modified Bradykinesia Rating Scale (13), alongside with the application of wearable technology (14). The motor subtypes are in fact characterized by a different progression, different NMS burden and different response to treatment, which might reflect a variable involvement of non-dopamine neurotransmitters (15–19).

### Clinical tests for motor

#### Imaging-based clinical tools

Presynaptic dopamine transporter (DaT) single-photon emission computerized tomography (DaTScan) reflects the loss of dopaminergic neurons in the substantia nigra (SN), measured as caudate, putamen and striatum binding ratios (20). DaTScan is a validated tool used to support the diagnosis of Parkinson’s and parkinsonism and in PD, it shows dopamine depletion starting posteriorly from the putamen (21). In PD, DaTScan can also correlates with the laterality of symptoms (22), and some studies suggest that the striatal binding ratio has a negative correlation with motor scores (23–25). Additionally, the tracer uptake is more reduced in the AR and PIGD subtypes compared to TD one, supporting the hypothesis that dopamine depletion in the basal ganglia is the main mechanism for this specific subtypes and providing the evidence for a better levodopa response compared to TD one (26–28). Disease progression models also support its role in predicting motor disease progression (24, 29, 30). However, application of the DaTScan in real clinical practice is more difficult, given its cost and availability limitations, and more evidence needs to be provided on its role in predicting disease trajectory or its utility in management of dopamine-replacing treatments.

A promising tool to investigate the motor vital, despite still under investigation, is brain magnetic resonance imaging (MRI) scan paradigms. It has been shown in fact that specific sequences, such as neuromelanin or susceptibility-weighted imaging and diffusion imaging of the SN, represent a good biomarker of disease progression and correlate with motor symptom burden (31–35). However, this remains investigational and not widely available for clinical application.

Finally, given its practicality, transcranial sonography of the SN, should be taken into consideration for future possible clinical applications, being its usefulness not only confined to the differential diagnosis among PD and controls. It has in fact being demonstrated that the SN hyperechogenecity correlates to motor disability and gait impairment in PD (36). However, evidence in its role in monitoring the motor vital are still poor, it is operator dependent, and not widely available for clinical application.

#### Blood and tissue-based clinical tools

Neurofilament light chain (NfL) levels, a marker of neuronal injury, can be found increased in cerebrospinal fluid (CSF), serum, and plasma of PwP compared with controls, and correlate to motor phenotypes in PD (37–39). Specifically, plasma NfL levels are found to be higher in PIGD subtype than in TD subtype (40, 41), whilst serum NfL levels have shown to correlate with motor severity (42). CSF, serum, and plasma alpha-synuclein levels have been investigated in several studies for its mapping of motor features in PD, confirming its role as a potential clinical tool for assessing motor severity (43–46). We feel therefore these may be important markers to consider in future, once its validity is widely established, with focus on plasma and serum-based tests over CSF for clinician’s ease of application.

#### Other clinical tools

Wearable technology (WT) is widely used in research settings for motor monitoring, especially in the more advanced stages of PD, characterized by the presence of motor fluctuations and more severe sleep disturbances (47, 48). Given the ability of WT to provide objective and continuous monitoring, it has the potential to assist clinicians in their assessments of PwP. In fact, studies have provided supporting evidence over WTs ability to collect reliable daily measurements of motor features (49), together with information on the effectiveness of levodopa treatment (50). However, its clinical application is limited at present, given lack of standardization across the several possible WT available and in development (47). Therefore, clinicians may choose to use WT in addition to their clinical-based management strategies.

## Dashboard vital: nonmotor

NMS in PD are ubiquitous, from prodromal to palliative stage (51). Based on data-driven analysis as well as observational case series-based data, a recent description of nonmotor subtypes or endophenotypes, such as Park-Cognitive and Park-Pain, has been suggested. These subtypes may be underpinned by specific dominant neurotransmitter dysfunctions, largely cholinergic, noradrenergic, and serotonergic, apart from the well-known dopaminergic one (3, 52, 53). A subtype-specific management has been proposed (53) and the nonmotor dashboard of vitals aims to reflect tests that can identify the different subtypes, to aid a dashboard-triggered personalized medicine.

### Clinical tests for nonmotor

#### Imaging-based clinical tools

DaTScans abnormalities have been associated with multiple NMS (54), but it is unclear whether this measurement could be used to assess the overall burden of NMS in PD. This might be due to the predominantly non-dopaminergic nature of many NMS, therefore would not be captured by dopaminergic imaging alone (55).

123I-metaiodobenzylguandine (MIBG) scintigraphy can be used to evaluate the postganglionic cardiac autonomic denervation, typical of PD (56). Recent evidence has supported the use of MIBG scintigraphy for the evaluation of the burden of autonomic dysfunction, as measured by the wash-out rate which seems to correlate with severity of orthostatic hypotension (57). However, the results have not been replicated in other studies (58). MIBG use in NMS remains investigational at present and requires careful consideration.

#### Blood and tissue-based clinical tools

Serum uric acid (UA, urate) has been explored in several studies, given its potential neuroprotective effect in PD and its inverse association with the risk of developing PD (59–61). Lower UA levels have been correlated with experiencing higher NMS burden, whilst higher UA levels seem to be more protective (62–64). However, conflicting data exists for UA application as a measure of NMS, and as such, it’s use clinically is currently not appropriate without further large-scale studies (65–67).

Serum homocysteine (Hcy) has been posed as a gender-specific biomarker for males developing PD dementia (PDD) and for disease progression (68, 69). In a recent data-driven study, using ‘severe’ subtype (motor, non-motor and cognitive domains) had higher levels of serum Hcy and C-reactive protein (CRP) compared to other, less severe, subtypes (70). However, levodopa, the gold standard of therapy in PD, has been shown to increase Hcy levels (71–73) and so the real validity of Hcy measurement remains questionable.

Among CSF biomarkers, those associated with the overall burden of NMS include β-amyloid 1–42 (Aβ_1–42_) and α-synuclein (74, 75). In particular, CSF Aβ_1–42_ has been found to be a significant predictor of longitudinal increase in NMS severity over 2 years in a cohort of over 400 patients from the Parkinson’s Progression Marker Initiative (PPMI) (74). On the other hand, in a study involving 46 PwP, CSF α-synuclein was inversely correlated with the total score of the NMS Scale (NMSS) (75). However studies of CSF and plasma Aβ_1–42_ and α-synuclein remain inconsistent (76–80). Among other suggested markers, CSF and plasma NfL increase can also be associated with cognitive symptoms of PD (81, 82).

#### Other clinical tools

WT has been shown to be useful in the evaluation of NMS, specifically daytime somnolence, the presence of RBD (83) and nocturnal movements (84). Additionally, measures of bradykinesia and dyskinesia as collected through the Parkinson’s KinetiGraph (PKG), have showed to be associated with gastrointestinal problems (85).

Additionally, modern technologies can also help in the assessment of cognition in PD. Several computerized cognitive batteries are in fact available to screen and investigate cognitive impairment, which can be also useful for patients’ remote assessment and digital intervention such as cognitive training (65, 66).

### Dashboard vital: visual, gut and oral health

#### Visual health

Visual abnormalities are particularly frequent in PwP, with an overall prevalence ranging from 78 to 82% (67, 86). Nevertheless, they are surprisingly often neglected in both clinical and research settings, leading to functional consequences, such as falls at night-time related to reduced night vision, road traffic accidents in PwP who drive, dry eyes causing ocular irritation and overall reduced QoL for patients (67, 87).

PwP suffer from a wide range of visual defects, from contrast insensitivity and reduced color discrimination, symptoms usually present since the early stages, to oculomotor disturbances, in particular convergence insufficiency and diplopia (88–90). The pathophysiology of visual abnormalities in PD is complex, and is in part due to the retinal dopamine depletion (91), the presence of a PD-related retinopathy (92, 93), alongside neuronal dysfunction in the visual cortex (94), and cortical or basal ganglia circuits dysfunction (95). Dry eyes can be caused by decreased blinking rate and decreased tear production due to autonomic dysfunction (96), while other symptoms can be related to the use of PD medication, such as the rare levodopa-induced ocular dyskinesia, iatrogenic hallucinations, or blurred vision induced by monoamine oxidase inhibitors and amantadine (97–99). Prolonged use of amantadine might also cause changes in corneal endothelium and oedema (100, 101).

#### Gut health

Gut health is strictly intertwined with the control of motor symptoms, NMS, and pathophysiology burden of the disease (102). Dysphagia, a crucial NMS in advanced PD, has significant impact on nutrition, hydration, and general health in PwP (7, 103) and is often not considered in earlier stages of PD. Constipation impacts the QoL and levodopa absorption in PwP (104). Additionally, intestinal pseudo-obstruction (paralytic ileus) can be one of the presentations of severe constipation in advanced PD, representing a medical emergency (105).

Another gut health-related factor impacting symptom control in PD is *Helicobacter pylori* (HP)-induced gastritis, which has an increased prevalence in PwP (104). Eradication of HP in PwP has been shown by several studies to improve levodopa response and motor performances (106), although a randomized placebo-controlled trial refutes these findings (107).

HP infection, coupled with decreased intestinal transit time, diminish the gastric acid production too, facilitating gut dysbiosis, including small intestinal bacterial overgrowth (SIBO) (108). SIBO is associated with poor motor function and could be a potential future target for disease modifying treatment (109). Other possible targets associated with gut dysbiosis, are the PD specific microbiome profiles (110, 111), whereby bacteria belonging to the Lachnospiraeceae family and faecalibactrium genus, are emerging as the most consistently altered gut microbiome in PwP (112).

#### Oral health

Due to the impairments caused by motor and NMS, PwP may have poor oral health and hygiene, with consequent increase of oral disease such as periodontitis and cavities (113), which, in turn, can increase the burden of some of the NMS (e.g., swallowing issues, pain, anxiety) and worsen QoL (114). Oral health may also play a role in the pathogenesis of PD, contributing via oral-dysbiosis, to induce systemic inflammation measurable, amongst others, with CRP (115–120).

Among oral bacteria, *Porphyromonas gingivalis* is associated with the presence of chronic periodontitis, and its toxic proteases, gingipains, and lipopolysaccharide, have been found in the blood of patients with PD (119), suggesting a possible pathological role in neurodegeneration (121, 122).

Sialorrhea, affecting up to 80% of PwP, often is the by-product of reduced swallowing, sealed lips from hypomimia, and poor awareness of drooling due to changes in sensation and posture (123, 124).

### Clinical tests for visual, gut, and oral health

#### Visual health

Numerous studies have shown significant retinal nerve fiber layer thinning and macular volume reduction at optical coherence tomography (OCT) in PwP compared to controls (125–127), yet there remains no agreement on which retinal segments are the most affected (125). These findings explain symptoms such as decreased visual acuity and impaired contrast sensitivity, altered color perception, and seem to be associated with disease stage and severity (125–127).

An earlier biomarker, even with normal OCT findings, is the bioelectrical retinal dysfunction shown with the electroretinography (ERG) and measured via several parameters, such as alpha-wave, beta-wave, or oscillatory potentials amplitude (128–130). Similarly, visual evoked potentials (VEP), can be used to discriminate PD patients from controls in early disease stages, being a higher P100 latency the most consistent biomarker (131).

Corneal confocal microscopy (CCM), measuring parameters like corneal nerve branch density, corneal nerve fiber length, corneal total branch density, and corneal nerve fiber area, is a non-invasive tool to evaluate small nerve and autonomic fibers damage (132). Several studies have demonstrated the presence of corneal nerve fiber pathology in PwP than controls (133–135), whose extension have been shown to be associated with disease severity, and specifically motor, autonomic and cognitive dysfunction (135–137).

Tear film tests, such as Schirmer’s test or tear film breakup time associated with dry eyes, together with blink rate evaluated with specific cameras, have been demonstrated to be abnormal in PwP and to correlate with disease severity (96, 138, 139). Furthermore, initial findings on tears protein composition, have shown a significant increase of oligomeric alpha synuclein in both basal and, more largely, reflex tears in PwP compared to healthy controls, paving the way for a new potential approach to alpha synuclein-based biomarkers (140), which need further exploration for clinical application.

Since nyctalopia (night-blindness) is the earliest and most common symptom of hypovitaminosis A (141), and PD might be associated to low levels of specific vitamins such as vitamin D and B12 (142, 143), the possibility of reduced blood and/or CSF concentrations of vitamin A (retinol) should be investigated. This deficit could contribute to worsen PD-related visual symptoms at the most challenging time of the day for a PwP, and therefore we believe it should be taken into consideration, as potentially reversible. However, studies have failed to show a systemic reduction of vitamin A levels in PwP, being its concentration controlled by a rigorous homeostatic process, or of retinoic acid, retinol’s active metabolite, because of its high biochemical instability (144).

#### Gut health

Upper gastrointestinal symptoms screening has been proposed for evaluating swallowing and oropharyngeal dysfunction (145). Diagnostic tools for swallowing abnormalities can be extensive, and include videofluoroscopic swallow study (VFSS) for the oropharyngeal phase of swallowing, or the fiberoptic endoscopic evaluation of swallowing (FEES) for the pharyngeal phase of swallowing, with both VFSS and FEES having a role in the identification of aspiration, particularly significant in the advance stages (146). From a practical and pragmatic point of view a VFSS seems to be the most relevant.

Constipation is a clinical diagnosis although imaging such as abdominal XR and CT scans can be used to demonstrate its severity. Radiological findings of constipation can include evidence of fecal loading throughout the colon, luminal faecalomas, with or without reduction of luminal gas (147). Furthermore, abdominal XR or CT helps in the diagnosis of pseudo-obstruction showing dilation of the colon, with or without other associated radiological features such as a volvulus-sign and is strongly recommended in advanced PD cases, particularly when screening for intrajejunal levodopa infusion therapies (105).

HP diagnostic testing are numerous, including the urea breath test, the stool antigen test, serology, and polymerase chain reaction, and should also be used to confirm the absence of HP post-eradication therapy (148). Gut dysbiosis and local proinflammatory status can be measured through sampling using stool microbiota (112), while the hydrogen breath test specifically applies to the identification of SIBO (149).

#### Oral health

For the effective evaluation of oral health in PwP, current recommendations advise for regular review by a dentist, with particular attention to the presence of local inflammatory conditions such as periodontitis (150).

Systemic inflammatory markers, such as CRP, might have a role in early detection of inflammatory oral conditions in PD, together with other systemic inflammatory markers, e.g., WBC, IL-1alpha, IL-1beta, IL-17A and TNF-alpha (118–120).

To identify *Porphyromonas gingivalis*, the possibility of detecting gingipains in the bloodstream, particularly gingipain R1, and the analysis of the oral microbiota through a saliva sample, should be explored further (116, 117, 119).

A simple salivatory test consisting in an Enzyme-linked immunosorbent assay (ELISA)-based multi-marker test of proteins including matrix metalloproteinases (MMP8, MMP9, MMP2, MMP3), has shown promising results in early diagnosis of gingivitis and periodontitis (125), but this remains under investigation.

Objective measurement of sialorrhea is often difficult, however methodologies exist, including salivation collection (151), suctioning (152), swallowing counts (153), Lashley cup over the parotid duct (153), and dental cotton buds in the mouth (154). However, these objective tests are often time consuming, costly, and poorly practicable in clinical practice.

## Dashboard vitals: bone health and falls

PwP are at increasing risk of developing osteoporosis, with women having a significantly higher risk than men with PD (155). In fact, the prevalence of osteoporosis has been observed in 91% of women and 61% of men with PD (155).

Osteoporosis is a reduction of bone mineral density (BMD) subsequently increasing the risk of fragility fractures (156). Several pathophysiological mechanisms seem to be implicated in the association between PD and osteoporosis and reduced BMD, and these include: female gender, vitamin D deficiency, reduced exposure to sunlight, low body weight, nutritional status, vitamin B12, folate deficiency and hyperhomocysteinemia, PD duration and severity, immobility, decreased muscle strength and other neuroendocrinal status (e.g., increased serum concentration of under-carboxylated osteocalcin) (155, 157).

Recent evidence has suggested that osteoporosis associated with PD might be caused by the dopaminergic degeneration itself, as well as by the treatment with levodopa, which could promote osteoclastogenesis and suppress bone formation, in association with elevated prolactin and following both gonadal steroid hormone-dependent and-independent metabolic pathways (158).

Considering the higher risk of falls due to postural and gait impairment in PwP (159–161), the presence of osteoporosis further increases the risk of fractures and subsequent hospitalization compared to age-matched individuals (8, 162).

### Clinical tests for bone health and falls

#### Imaging-based clinical tools

To confirm the diagnosis of osteoporosis, a dual-energy X-ray absorptiometry (DEXA) at the hip is required: according to the World Health Organization classification, a BMD of 2.5 standard deviations (SD) below that of a young adult defines osteoporosis, while value on DEXA between 1 and 2.5 SD is considered as osteopenia (157). In our opinion, DEXA should be obligatory in PD patients over the age of 50.

Recently, other imaging techniques have been investigated in PD, such as distal radius DEXA, which could potentially optimize the detection of osteoporosis, especially in female PD patients (163), and the less invasive calcaneal quantitative ultrasound to assess bone quality, which has demonstrated good correlation with DEXA measurements and has been recently evaluated in a cohort of PwP (164).

#### Blood-based clinical tools

To promptly address and, when possible, prevent osteoporosis in PwP, several simple blood tests can be undertaken to get an overview of the calcium metabolism and other potential risk factors for osteoporosis. First and foremost, serum levels of 25-OH-vitamin D have been shown to directly correlate with BMD of the hip and lumbar spine in PD (155, 165, 166). Additionally, serum ionized calcium has also been found to correlate with BMD and to be significantly different between PwP and healthy controls (166).

#### Other clinical tools

The Fracture Risk Assessment Tool (FRAX) (167) helps predict the 10-year probability of hip fractures and major osteoporosis-related fractures, and has been used in several large studies exploring osteoporosis and fracture risks in the population (156). FRAX tool may require adjustment to have PD as an independent risk factor for fractures and as such, requires a modern adaptation to PwP (168, 169).

Wearable sensors have been shown to be reliable in PwP for detecting balance issues and predict risk of falls. WT can analyze gait and balance in real-time whilst providing feedback (e.g., visual, and auditory cues) for features of postural instability, such as freezing of gait. This feedback provided by WT can then reduce the risk of fall events from occurring, as such, WT can be a promising tool for rehabilitation and falls prevention (170, 171).

## Dashboard vital: co-morbidities, co-medications, and dopamine agonists side effects

Several global epidemiological studies confirmed the present of several comorbidities in PwP, which can be grouped as cardiovascular (e.g., hypertension, heart failure), neuropsychiatric (e.g., depression, dementia), and others including diabetes mellitus, arthritis, and urinary symptoms (172–177). Advanced stages of PD are associated with balance issues and postural instability (178). Despite no evidence of direct involvement of the vestibular system in PD, a possible vestibular hypofunction could be considered in patients with dizziness but not orthostatic hypotension (179). The importance of appreciating PwP co-morbidities in their management has been highlighted by Schrag and colleagues (2022) in a case–control study, observing how certain co-morbidities and risk factors may reflect potential early extra-striatal and extracerebral pathophysiology in PwP (180).

Given the progressive nature of PD, co-medications, particularly in the advanced stages, represent a clinical challenge and expose patients to increased risk for drug interaction and hospitalization (181, 182). Additionally, the required increased dopaminergic stimulation in PD, in particularly when using dopamine agonists alone or in combination, might give emergence to significant side effects, such as severe daytime sleepiness (183) or impulse control disorders (ICDs) (184). ICDs and impulse control behaviors encompass a range of presentations including hypersexuality, gambling, binge eating, and dopamine dysregulation syndrome (185).

### Clinical tests for co-morbidities, co-medications, and dopamine agonists side effects

#### Co-morbidities

Studies report that there is an association between diabetes and PD progression, with a greater motor burden and motor decline been demonstrated in PwP who have diabetes (186, 187). Non-diabetic hyperglycaemia, with recorded glycosylated hemoglobin (HBA1c) levels above 45 mmol/mol, is an independent predictor of quicker motor progression (188–190).

The MARK-PD Study evaluated the relationship between diabetes, HBA1c and serum NfL levels as markers of neuroaxonal damage in PwP, concluding that those with diabetes and PD had higher serum NfL and more cognitive impairment, with diabetes also associated with having a higher H&Y score (191, 192).

Weight variability is a common clinical finding in PwP and yet remains poorly understood (193). Unexplained weight loss has been reported across all stages of PD (193) and it has also been proposed as a prodromal feature (194). Monitoring the patient’s body mass index (BMI) can be a simple and effective way to prevent pathological weight loss leading to poorer prognosis and higher mortality (195, 196). Weight loss in PD has also been associated with faster cognitive decline, while presynaptic dopaminergic depletion in the right striatum may serve as a predictor of future weight changes (197).

Blood pressure fluctuations have been recognized in PD, due to the concomitant involvement of the autonomic system (198), which can present as postprandial hypotensive episodes during the day, as well as nocturnal hypotension, causing great limitation of QoL. These fluctuations can be measured using a 24-h ambulatory blood pressure monitoring device (199). Further assessment through the head-up tilt-test gives clinicians insight into the activation of the autonomic systemic compensatory mechanism in PwP (200).

Dizziness, in the absence of orthostatic hypotension, can be examined using the Vestibular Evoked Myogenic Potentials, to consider vestibular hypofunction as a cause (179). However, the clinical application of this remains under investigation.

#### Co-medications

Co-morbidities Polypharmacy Score (CPS) is a simple clinical tool providing a surrogate measure of co-morbidities burden, correlating with poor outcomes and mortality rates (201, 202). When assessing co-medications, particular attention should be given to the anticholinergic burden, especially in the older adults, considering the numerous medications that have anticholinergic properties, such as analgesics, antispasmodics, antiarrhythmics and anti-Parkinson’s (203). Anticholinergic burden has been linked to functional and cognitive decline in PwP, with possible contribution to overt dementia, and potential side effects including constipation, agitation, urinary retention, and delirium (203, 204). The use of anticholinergic indexes, such as Anticholinergic Drug Scale (ADS) and the Drug Burden Index - Anticholinergic Component (DBI-ACh), are useful clinical tool for such assessments, with ADS being shown to correlate with serum anticholinergic activity (203, 205, 206).

#### Dopamine agonist side effects

For ICDs, the use of electroencephalography to measure feedback-related negativity has been suggested to reflect the reward-processing of individuals, thus potentially representing a predictive clinical tool (207, 208).

Excessive daytime sleepiness (EDS) is a known side effect of dopamine agonists, with higher D2/D3 receptor agonism, such as Ropinirole and Pramipexole (183, 209, 210), and can be assessed with Multiple Sleep Latency Tests (MSLT) (211, 212). If EDS presents in the absence or withdrawal of dopamine agonists intake, then obstructive sleep apnoea should be suspected and polysomnography should be considered as further investigation (213, 214).

## Conclusion

We suggest the use of ‘The Chaudhuri’s Dashboard of Vitals for Parkinson’s’ for the care of PwP. This tool can incorporate several clinical tests that might help clinicians with the screening, diagnosis, treatment, and monitoring of key and potentially health threatening factors. Until now, only two of the five vitals have been widely investigated in clinical practice, namely motor and nonmotor assessments. In this paper, we have worked to highlight the importance of three other vitals (Vital 3–5, Figure 1), which have an impact on not only the QoL for PwP, but also the disease progression and prognosis. Some clinical tests discussed in this paper are already available in either general or PD-related clinical practice, whilst others have the potential of future implementation, providing they are validated and are cost-effective for healthcare systems across the globe.

## Author contributions

MQ, SR, LB, IS, CF-P, NT, and KR contributed toward the design and methodology. MQ, SR, LB, and KR contributed toward the formal analysis and first draft of the manuscript. All authors contributed toward the conceptualization, review, and editing of the manuscript for submission.

## Conflict of interest

The authors declare that the research was conducted in the absence of any commercial or financial relationships that could be construed as a potential conflict of interest.

## Publisher’s note

All claims expressed in this article are solely those of the authors and do not necessarily represent those of their affiliated organizations, or those of the publisher, the editors and the reviewers. Any product that may be evaluated in this article, or claim that may be made by its manufacturer, is not guaranteed or endorsed by the publisher.
